# Ion concentration dynamics leads to the very slow spontaneous neuronal oscillations

**DOI:** 10.1186/1471-2202-15-S1-P217

**Published:** 2014-07-21

**Authors:** Giri P Krishnan, Oscar Gonzales, Maxim Bazhenov

**Affiliations:** 1 Department of Cell Biology & Neuroscience, University of California, Riverside, CA, USA

## 

Resting or baseline brain activity may fluctuate at the very low frequencies (0.01-0.1 Hz) [1]. These oscillations, revealed by fMRI data, are found to be correlated across brain regions. There has been rising interest to this slow activity, since it may reflect functional connectivity between brain regions and strongly correlate with neuronal firing.

In this study we propose that spontaneous slow brain oscillations may rise from ionic concentration dynamics. We used biophysically realistic computational model of the cortical network based on the Hodgkin-Huxley kinetics and implementing ion concentration dynamics. Computational network model consisted of pyramidal and inhibitory neurons with connectivity based on AMPA, GABAa and NMDA synapses. It implemented dynamic variables for intra- and extracellular K+/Na+ concentrations, intracellular Cl- concentration and simulated Na+/K+ exchange and KCC2 pumps. Random Poisson input was given to each neuron to mimic in vivo conditions.

Our study revealed that extracellular K+ dynamics determine the slow network oscillations in the frequency range 0.01-0.05 Hz. The network oscillations were more prominent for larger network compared to the small networks. The oscillation amplitude depended on the random input to the network. While K+ dynamics and diffusion contributed to the local synchrony, long-range synaptic connectivity provided correlated activity between distinct populations of neurons with no ion diffusion between sites. This finding may explain observation that structural network connections determine the functional connectivity in human recordings [2]. We conclude that the ion concentration dynamics may contribute to the slow spontaneous fluctuations in brain activity found with fMRI recordings. Future experiments are required to verify this prediction.
